# A modified in vitro clot lysis assay predicts outcomes and safety in acute ischemic stroke patients undergoing intravenous thrombolysis

**DOI:** 10.1038/s41598-021-92041-1

**Published:** 2021-06-16

**Authors:** Rita Orbán-Kálmándi, István Szegedi, Ferenc Sarkady, István Fekete, Klára Fekete, Nikolett Vasas, Ervin Berényi, László Csiba, Zsuzsa Bagoly

**Affiliations:** 1 Division of Clinical Laboratory Sciences, Department of Laboratory Medicine, Faculty of Medicine, Kálmán Laki Doctoral School, University of Debrecen, 98 Nagyerdei krt., Debrecen, 4032 Hungary; 2 Department of Neurology, Faculty of Medicine, University of Debrecen, 22 Móricz Zsigmond krt., Debrecen, 4032 Hungary; 3 Department of Radiology, Faculty of Medicine, University of Debrecen, 98 Nagyerdei krt., Debrecen, 4032 Hungary; 4 ELKH-DE Cerebrovascular and Neurodegenerative Research Group, 22 Móricz Zsigmond krt., Debrecen, 4032 Hungary

**Keywords:** Neurological disorders, Stroke, Predictive markers

## Abstract

The outcome of intravenous thrombolysis using recombinant tissue plasminogen activator (rt-PA) is only favorable in ≈ 40% of acute ischemic stroke (AIS) patients. Moreover, in ≈ 6–8% of cases, intracerebral hemorrhage (ICH) develops. We tested whether a modification of clot lysis assay (CLA), might predict therapy outcomes and safety. In this prospective observational study, blood samples of 231 AIS patients, all receiving intravenous rt-PA, were taken before thrombolysis. Cell-free DNA (cfDNA), CLA and CLA supplemented with cfDNA and histones (mCLA) were determined from the blood samples. Stroke severity was determined by NIHSS on admission. ICH was classified according to ECASSII. Short- and long-term outcomes were defined at 7 and 90 days post-event according to ΔNIHSS and by the modified Rankin Scale, respectively. Stroke severity demonstrated a step-wise positive association with cfDNA levels, while a negative association was found with the time to reach 50% lysis (50%CLT) parameter of CLA and mCLA. ROC analysis showed improved diagnostic performance of the mCLA. Logistic regression analysis proved that 50%CLT is a predictor of short-term therapy failure, while the AUC parameter predicts ICH occurrence. A modified CLA, supplemented with cfDNA and histones, might be a promising tool to predict short-term AIS outcomes and post-lysis ICH.

## Introduction

Acute ischemic stroke (AIS) is a leading cause of death and adult disability in all developed countries^1,2^. As of today, two major causal treatments of AIS are approved: intravenous thrombolysis using recombinant tissue plasminogen activator (rt-PA) and endovascular treatment (mechanical thrombectomy), both targeting rapid recanalization of the blocked vessel^3^. Unfortunately, these therapies are not a remedy for all, as endovascular treatments are not widely available and thrombolysis is limited by its narrow therapeutic time window^4^. Moreover, although the benefit of thrombolysis using rt-PA within 4.5 h after the onset of symptoms has been proven, only ≈ 40% of treated patients show improvement, while recanalization fails in most cases^3,5,6^. On the other hand, in approximately 6–8% of patients, intracranial bleeding develops as a potentially life-threatening side-effect^7^. In selected patients, thrombolysis and mechanical thrombectomy can be performed in conjunction, but the risk/benefit profile of intravenous thrombolysis prior to mechanical thrombectomy is often debated^8^. Despite all efforts to invent prediction models for patient subgroups that are more likely to benefit or not benefit from intravenous thrombolysis, major contributors, which influence lysis susceptibility and have a direct clinical impact on outcomes and safety remain to be determined^9^. It can be surmised that individual factors altering clot structure and lysis susceptibility are likely to be key contributors to recanalization failure and bleeding complications. As the number of these contributors can be many, global assays of in vitro clot formation and lysis, such as the in vitro clot lysis assay (CLA), might prove useful when it comes to testing patients in the acute clinical setting.

Studies on thrombus composition retrieved from cerebral arteries have shown that neutrophil extracellular traps (NETs) are important constituents of ischemic stroke thrombi^10,11^. NETs are fibrous networks of extracellular DNA, histones, and neutrophil granule proteins that have been first implicated in host defense as part of the innate immune system over a decade ago^12^. In recent animal and human studies, NETs have been proposed to contribute to the pathogenesis of thrombotic disorders, including stroke, by various prothrombotic and antifibrinolytic effects^13–19^. It has been shown that NETs intercalate to fibrin and create a dense network that is resistant to fibrinolysis^17,20,21^. Inter-individual differences of such effect in patient cohorts have not been investigated, as yet. Recently, it has been shown that increased thrombus cell free DNA (cfDNA) content decreases the efficacy of rt-PA treatment, and a strategy involving the administration of deoxyribonuclease 1 (DNAse 1) in addition to thrombolysis has been proposed^11^. Before implementing such approaches in the clinical practice, further research is warranted. Circulating cfDNA levels, in theory could be associated with thrombus cfDNA content, moreover, cfDNA has been proposed as a potential biomarker that could be used to predict the efficacy of rt-PA and guide treatment decisions^22^. Furthermore, supplementation of clot lysis tests with cfDNA and histones to better imitate conditions present in AIS thrombi may directly reflect lytic susceptibility of thrombi and thus neurologic outcomes, however, today no hemostasis test exists that takes the effect of NET components into consideration.

The aim of this study was to evaluate the levels of cfDNA in a relatively large cohort of AIS patients before thrombolysis, to test the effect of cfDNA on in vitro CLA and to evaluate the association with clinical outcomes. We also aimed to find out whether a modified in vitro CLA, that incorporates the effect of cfDNA and histones, potentially present within the thrombus, might better predict therapy outcomes and safety as compared to the conventional assay.

## Results

A total of 231 AIS patients receiving intravenous thrombolysis with rt-PA according to standard protocols were included in the study. Baseline characteristics of patients and stroke outcomes are shown in Table 1. Median age of the cohort was 67 (IQR: 50–76) years, 54.6% were men. Median NIHSS on admission was 7 (IQR: 4–11). Median time from symptom onset to treatment with rt-PA was 150 (IQR: 111–206) min. Favorable short- and long-term outcome was achieved in 41.9% and 45.0% of patients, respectively. Intracerebral bleeding occurred in 18 patients (7.8%), in 6 cases it was symptomatic.

**Table 1:** Baseline characteristics and outcome of enrolled patients.

Number of patients	231
Age, y	67 (50–76)
Male sex, n (%)	125 (54.6)
Stroke severity on admission, NIHSS	7 (4–11)
**Cerebrovascular risk factors, n (%)**	
Arterial hypertension	188 (81.4)
Atrial fibrillation	43 (18.6)
Diabetes mellitus	61 (26.4)
Hyperlipidemia	150 (64.9)
Active smoker	65 (28.1)
Previous stroke, TIA	56 (24.2)
BMI, kg/m^2^	28.3 (± 6.3)
**Medication at enrollment, n (%)**	
Antihypertensive therapy	118 (51.0)
Antiplatelet drug	87 (37.7)
Anticoagulant drug	15 (6.5)
Lipid-lowering therapy	56 (24.2)
Antidiabetic therapy	36 (15.6)
**Laboratory measurements on admission**	
INR	0.99 (0.94–1.04)
APTT, sec	27.4 (25.6–29.9)
WBC count, G/L	8.14 (6.4–10.3)
Platelet count, G/L	227 (179–265)
Serum glucose, mmol/L	6.6 (5.8–8.2)
hsCRP, mg/L	2.9 (1.6–7.3)
Creatinine, μmol/L	74 (63–90)
Fibrinogen, g/L	4.2 (3.5–4.8)
D-dimer, mg/L	0.79 (0.50–1.47)
Plasminogen activity, %	97 (87–110)
α2-plasmin inhibitor activity, %	103 (92–110)
**Stroke etiology (TOAST), n (%)**	
Large-artery atherosclerosis	90 (39.0)
Small-vessel occlusion	38 (16.5)
Cardioembolic	30 (13.0)
Other/undetermined	73 (31.5)
**Imaging data, n (%)**	
ASPECTS on admission	
0–7	4 (2.2)
8–10	179 (97.8)
ASPECTS at 24 h after thrombolysis	
0–7	30 (16.6)
8–10	151 (83.4)
**Thrombolysis (i.v. rt-PA) treatment**	
Duration of thrombolysis, min	62 (± 12)
Time from symptom onset to treatment, min	150 (111–206)
rt-PA dose, mg	66.3 (± 18.1)
**Outcomes, n (%)**	
Short-term outcome (∆ NIHSS by day 7)^a^	
Favorable (−4 points or NIHSS = 0 by day 7)	97 (41.9)
Unchanged status (± 3 points)	78 (33.8)
Unfavorable (+ 4 points or more)	17 (7.4)
Undetermined	21 (9.1)
Long-term outcome (mRS, day 90)^a^	
Favorable (mRS 0–1)	104 (45.0)
Unfavorable (mRS 2–6)	89 (38.5)
Undetermined	20 (8.7)
Intracerebral hemorrhage, ICH (ECASS II, 24 h)	
No ICH	213 (92.2)
aSICH	12 (5.2)
SICH	6 (2.6)

Data are means ± SD or medians (interquartile ranges).

aSICH, asymptomatic intracerebral hemorrhage; APTT, activated partial thromboplastin time; ASPECTS, Alberta Stroke Program Early CT Score; BMI, body mass index; ECASS II, European Co-operative Acute Stroke Study-II; hsCRP, high sensitivity C-reactive protein measurement; ICH, intracerebral hemorrhage; INR, international normalized ratio; i.v., intravenous; mRS, modified Rankin Scale; n, number of patients; NIHSS, National Institutes of Health Stroke Scale; rt-PA, recombinant tissue plasminogen activator; SICH, symptomatic intracerebral hemorrhage; TIA, transient ischemic attack; TOAST, Trial of ORG 10172 in Acute Stroke Treatment; WBC, white blood cell. Baseline NIHSS was not available in case of 2 patients.

^a^Excluding patients with therapy-associated ICH.

### Cell free DNA (cfDNA) levels, AIS severity and outcomes

**Figure 1:**
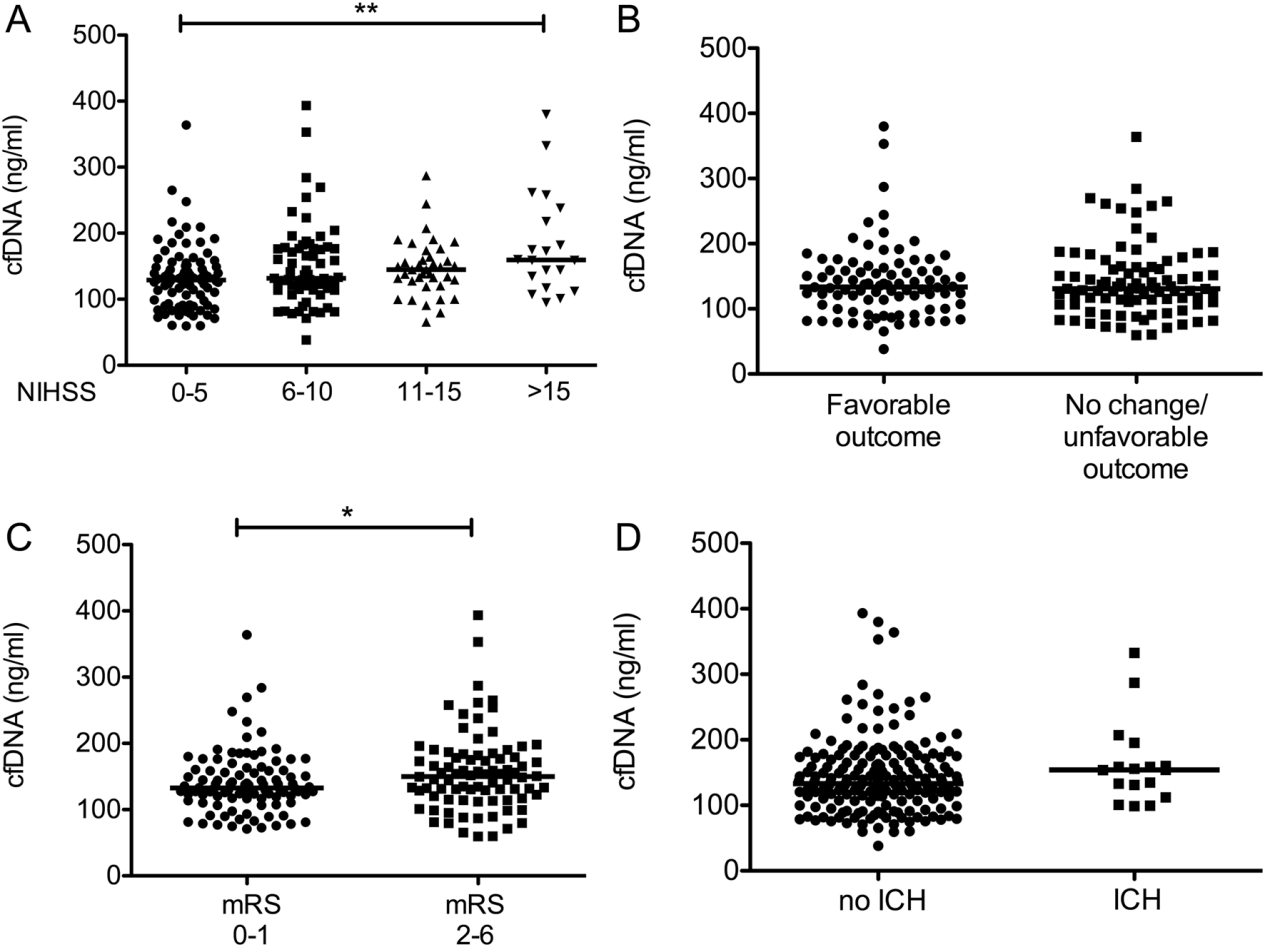
Association between cell free DNA (cfDNA) levels on admission and the severity and outcomes of stroke. Association between cfDNA levels on admission and stroke severity (**A**), short term outcome of stroke (**B**), long-term outcome of stroke (**C**), and therapy-associated intracerebral hemorrhage (**D**). cfDNA, cell free DNA; ICH, intracerebral hemorrhage; NIHSS, National Institutes of Health Stroke Scale, mRS, modified Rankin Scale, **p < 0.01, *p < 0.05 [(**A**) Kruskal–Wallis with Dunn-Bonferroni post hoc test, (**B**–**D**) Mann–Whitney U test].

We examined the relationship between pre-thrombolysis cfDNA levels, AIS severity and outcomes. Stroke severity on admission demonstrated a step-wise association with cfDNA levels, patients with more severe stroke had significantly higher cfDNA levels as compared to patients with milder strokes (Fig. 1A). Stroke etiology according to TOAST classification did not show an association with cfDNA levels (data not shown). cfDNA levels on admission did not differ in patients with favorable or unfavorable short term outcomes of stroke (Fig. 1B). On the other hand, patients with favourable long-term outcomes of stroke (mRS 0–1) demonstrated significantly lower on admission cfDNA levels as compared to those with unfavourable outcomes at 90 days post-event (Fig. 1C). Admission cfDNA levels in patients with therapy-associated intracerebral hemorrhage did not differ significantly from the results of those without such complication (Fig. 1D).

### Association of clot lysis assay (CLA) parameters with cfDNA levels, AIS severity and outcomes

**Figure 2:**
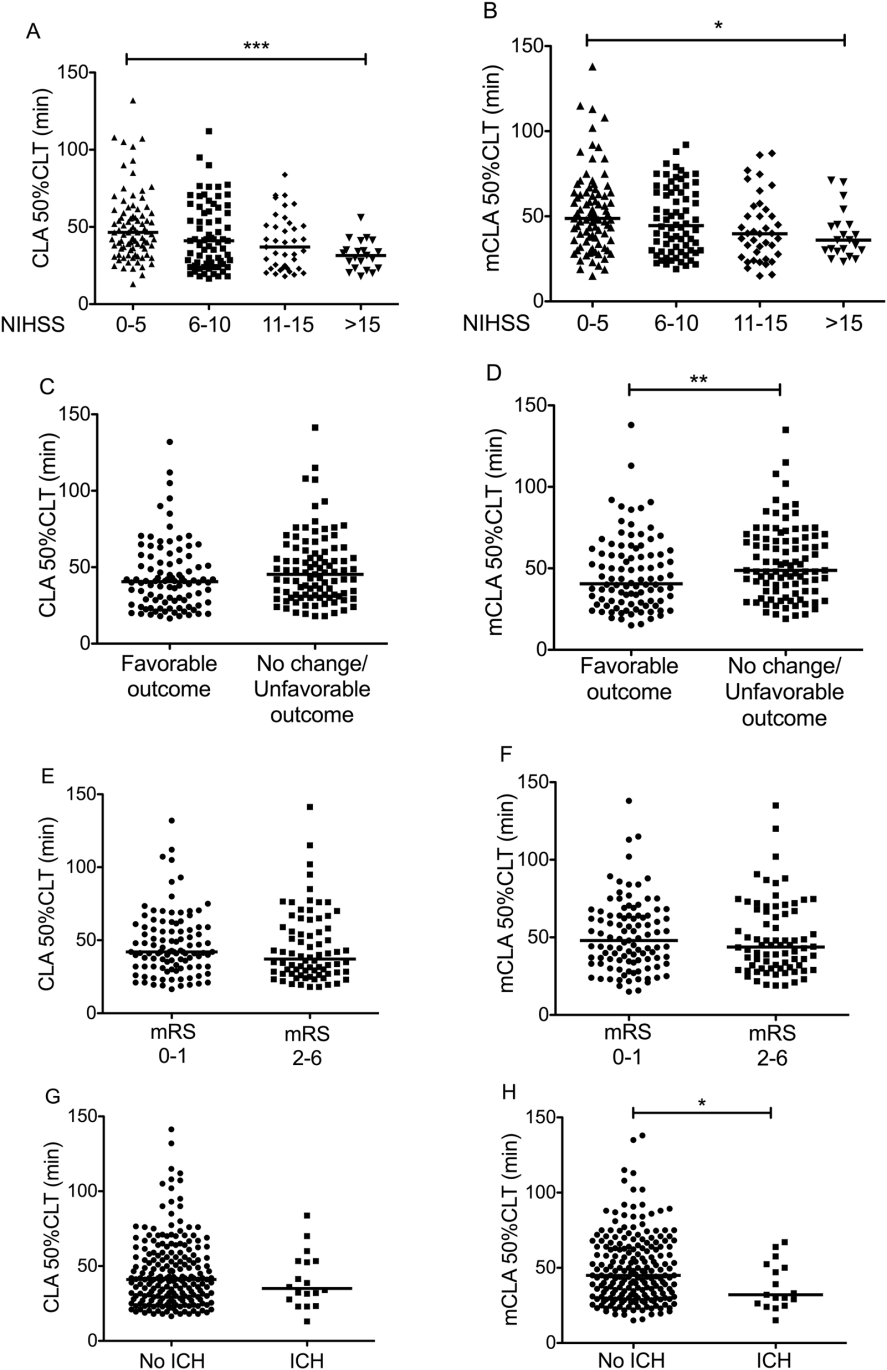
Association between 50% clot lysis time (50%CLT) parameter of the clot lysis assay (CLA) or modified clot lysis assay (mCLA) and the severity and outcomes of stroke. Association between the 50%CLT parameter of CLA and stroke severity (**A**), short term outcome of stroke (**C**), long-term outcome of stroke (**E**), and therapy-associated intracerebral hemorrhage (**G**). Association between the 50%CLT parameter of mCLA and stroke severity (**B**), short term outcome of stroke (**D**), long-term outcome of stroke (**F**), and therapy-associated intracerebral hemorrhage (**H**). CLA, clot lysis assay; mCLA, modified clot lysis assay including the effect of cfDNA and histones; 50% CLT, 50% clot lysis time, ICH, intracerebral hemorrhage; NIHSS, National Institutes of Health Stroke Scale, mRS, modified Rankin Scale. *p < 0.05, **p < 0.01, ***p < 0.001 [(**A**,**B**) Kruskal–Wallis with Dunn-Bonferroni post hoc test, (**C**–**H**) Mann–Whitney U test].

In this cohort, stroke severity on admission demonstrated a step-wise association with 50%CLT parameter (Fig. 2A), AIS stroke patients who suffered more severe stroke on admission presented significantly shorter 50%CLT. Similar significant associations were found in case of 10%CLT and CLA AUC parameters (Table 2). Despite a similar step-wise but inverse association of 50%CLT with stroke severity as seen in case of cfDNA and stroke severity, surprisingly, 50%CLT and cfDNA parameters did not show a significant correlation (Spearman r = 0.072; 95%CI: − 0.069 to 0.209, p = 0.301). Moreover, CLA parameters did not show any association with short- or long-term outcomes (Fig. 2C,E, and Suppl Table S1 and S2). We also found no association between bleeding complications and the 50%CLT parameter (Fig. 2G), while CLA AUC was significantly lower in patients experiencing post-lysis ICH (Table 3). Stroke etiology according to TOAST classification, treatment specifications including the time from symptom-onset-to-treatment and radiological severity of strokes based on ASPECTS (0 h and 24 h) did not show any association with CLA results (data not shown).

**Table 2:** Admission clot lysis assay parameters and specific hemostasis/fibrinolysis protein levels according to stroke severity (NIHSS) on admission.

	NIHSS 0–5 (n = 93)	NIHSS 6–10 (n = 72)	NIHSS 11–15 (n = 42)	NIHSS > 15 (n = 22)	p
**Clot lysis assay (CLA)**					
Maximal absorbance	1.37 (1.26–1.51)	1.43 (1.32–1.53)	1.38 (1.23–1.53)	1.36 (1.17–1.52)	0.282
Time to maximal absorbance (min)	12 (8.5–17.0)	10.0 (9.0–15.0)	10.0 (8.0–16.0)	11.0 (6.5–14.0)	0.783
10%CLT (min)	32.0 (23.5–43.5)	32.0 (19.0–42.5)	29.5 (19.0–40.0)	25.0 (19.0–32.0)	0.038
50%CLT (min)	46.5 (36.0–59.0)	41.0 (26.0–62.0)	37.0 (23.5–51.0)	31.5 (23.5–38.0)	0.006 0.0009*
90%CLT (min)	81.0 (62.0–111.5)	78.5 (59.0–109.0)	70.0 (51.5–92.0)	72.0 (55.0–94.0)	0.224
CLA AUC (OD * min)	27.41 (22.63–33.04)	27.68 (22.60–33.40)	24.70 (20.32–29.34)	24.25 (20.43–27.73)	0.024
**Clot lysis assay in the presence of cell-free DNA and histones (modified CLA)**					
Maximal absorbance	1.41 (1.32–1.51)	1.47 (1.35–1.57)	1.41 (1.29–1.56)	1.40 (1.19–1.55)	0.262
Time to maximal absorbance, (min)	11.0 (9.0–18.0)	10.0 (7.5–19.0)	11.0 (9.0–18.0)	12.0 (7.5–16.5)	0.956
10%CLT, (min)	35 (25.5–50.5)	38.0 (23.5–52.5)	30.5 (20.0–40.5)	27.5 (20.5–37.5)	0.042
50%CLT, (min)	49.0 (36.0–64.0)	45.5 (30.0–67.0)	40.0 (26.0–54.0)	36.0 (30.0–45.0)	0.018
90%CLT, (min)	85.0 (66.5–108.0)	84.5 (64.0–117.0)	75.5 (54.0–88.0)	81.5 (63.5–97.0)	0.167
CLA AUC, (OD * min)	28.18 (22.83–34.74)	29.91 (24.70–36.88)	26.58 (21.95–30.97)	25.84 (21.74–29.71)	0.023
**Specific hemostasis/fibrinolysis proteins**					
Fibrinogen (g/L)	4.0 (3.3–4.7)	4.3 (3.7–4.9)	4.2 (3.6–4.7)	4.1 (3.5–4.6)	0.101
D-dimer (mg/L)	0.7 (0.5–1.3)	0.8 (0.5–1.5)	0.9 (0.5–1.6)	1.3 (0.6–2.2)	0.158
Plasminogen activity (%)	101.0 (90.0–113.0)	98.0 (89.0–110.0)	92.0 (84.0–107.0)	87.0 (81.0–96.0)	0.011 0.017^*^
α2-PI activity (%)	103.0 (92.0–112.0)	104.0 (92.0–110.0)	103.0 (93.0–109.0)	98.0 (85.0–106.0)	0.254

Data are medians (interquartile ranges). NIHSS, National Institutes of Health Stroke Scale; 10%CLT, 10% clot-lysis time; 50%CLT, 50% clot-lysis time; 90%CLT, 90% clot-lysis time; CLA AUC, clot lysis assay area under the curve; *NIHSS 0–5 vs. NIHSS > 15 (Kruskal–Wallis with Dunn-Bonferroni post-hoc test). Baseline NIHSS was not available in case of 2 patients.

**Table 3:** Admission clot lysis assay (CLA) parameters and specific hemostasis/fibrinolysis protein levels according to the absence or presence of post-thrombolysis intracranial bleeding complications.

	No bleeding (n = 213)	ICH (n = 18)	p
**CLA**			
Maximal absorbance	1.4 ± 0.2	1.4 ± 0.2	0.361
Time to maximal absorbance (min)	10.5 (8.8–15.0)	12.3 (7.5–16.7)	0.866
10%CLT (min)	30.0 (19.5–40.0)	25.3 (19.1–42.9)	0.524
50%CLT (min)	41.0 (28.9–57.5)	35.0 (26.6–53.3)	0.396
90%CLT (min)	77.6 (57.9–108.0)	81.8 (53.0–108.0)	0.496
CLA AUC (OD * min)	26.3 (22.1–32.4)	23.8 (18.3–27.3)	0.028
**CLA in the presence of cfDNA and histones (modified CLA)**			
Maximal absorbance	1.4 ± 0.2	1.4 ± 0.2	0.149
Time to maximal absorbance, (min)	11.3 (9.0–18.0)	12.4 (6.9–19.0)	0.892
10%CLT, (min)	33.0 (23.0–49.0)	24.3 (18.6–41.6)	0.111
50%CLT, (min)	45.0 (30.9–64.0)	32.5 (25.9–50.6)	0.034
90%CLT, (min)	84.0 (65.0–108.0)	81.8 (53.0–108.0)	0.512
CLA AUC, (OD * min)	28.3 (23.2–34.3)	24.1 (20.2–31.7)	0.005
**Specific hemostasis/fibrinolysis proteins**			
Fibrinogen (g/L)	4.1 (3.5–4.7)	4.5 (3.8–5.1)	0.167
D-dimer (mg/L)	0.8 (0.5–1.4)	1.5 (0.6–2.4)	0.029
Plasminogen activity (%)	97.0 (88.0–110.0)	89.0 (81.0–99.0)	0.023
α2-PI activity (%)	102.0 (92.0–110.0)	105.0 (96.0–108.0)	0.979

Data are means ± SD or medians (interquartile ranges).

α2-PI, α2-plasmin inhibitor; cfDNA, cell-free DNA; CLA, clot lysis assay; 10%CLT, 10% clot lysis time; 50%CLT, 50% clot lysis time; 90%CLT, 90% clot lysis time; CLA AUC, clot lysis assay area under the curve; ICH, intracerebral hemorrhage; n, number of patients.

### Association of the modified clot lysis assay (mCLA) parameters with AIS severity and outcomes

In order to better imitate conditions present in AIS thrombi, we modified the CLA by adding cfDNA and histones in access to the clot induction and lysis mixtures (modified CLA). Concentrations of cfDNA and histones used in the assay were selected based on their presumed concentration within the thrombi according to previous literature and based on biochemical studies where the combined effect of histones and cfDNA were studied on fibrinolysis kinetics in purified experimental conditions^21^. As expected based on previous reports using purified proteins^20^, the presence of cfDNA and histones affected clot formation and prolonged clot lysis significantly in the total cohort (Table 4). Median time to reach 50% clot lysis was delayed by 4 min when cfDNA and histones were present in the assay mixture. Similary to the conventional CLA assay, stroke severity on admission demonstrated a step-wise association with 50%CLT parameter of the modified assay (Fig. 2B), and similar significant associations were found in case of 10%CLT and CLA AUC parameters of mCLA (Table 2). Notably, also in the case of mCLA, patients with more severe stroke presented significantly shorter clot lysis. In order to explain this finding, specific hemostasis proteins suggestive of consumption or excessive fibrinolysis were measured from all samples (Table 2). While fibrinogen levels did not suggest extensive consumption related to stroke severity, interestingly, plasminogen activity showed a significant step-wise decrease in case of more severe strokes. This finding, however, does not explain shorter clot lysis in case of more severe strokes. Although it did not reach the level of significance, a decreasing trend was observed for α2-PI activity in case of most severe strokes (NIHSS > 15).

**Table 4:** Admission clot lysis assay (CLA) parameters in the absence or presence of cell-free DNA (cfDNA) and histones in the total cohort.

	CLA	CLA in the presence of cfDNA and histones (modified CLA)	p
Max. absorbance (OD)	1.39 (± 0.20)	1.42 (± 0.19)	< 0.0001
Time to max. absorbance (min)	11.0 (8.3–15.0)	11.3 (9.0–18.0)	0.0018
10%CLT (min)	30.0 (19.5–40.5)	32.3 (22.0–49.0)	< 0.0001
50%CLT (min)	41.0 (29.0–57.0)	45.0 (30.0–64.0)	< 0.0001
90%CLT (min)	76.0 (57.0–108.0)	83.0 (65.0–108.0)	< 0.0001
CLA AUC (OD * min)	26.1 (21.7–32.1)	27.8 (22.8–33.7)	< 0.0001

Data are means ± SD or medians (interquartile ranges).

cfDNA, cell-free DNA; CLA, clot lysis assay, 10%CLT, 10% clot lysis time; 50%CLT, 50% clot lysis time; 90%CLT, 90% clot lysis time; CLA AUC, clot lysis assay area under the curve.

As compared to those who did not benefit from thrombolysis, patients with favorable short-term outcomes showed significantly shorter 50%CLT in the mCLA (Fig. 2D). This association was not significant in case of the original assay when cfDNA and histones were not present in the assay mixture (Fig. 2C). Long-term outcomes, however, showed no association with mCLA results (Fig. 2F). The occurrence of therapy-associated intracerebral hemorrhage showed a significant association with clot lysis parameters, particularly when the assay was modified using cfDNA and histones (Fig. 2H, Table 3). In patients who suffered post-lysis intracerebral bleeding, 50%CLT was significantly shorter in the modified assay as compared to those without such complications. No difference was observed between patients experiencing symptomatic hemorrhage as compared to those with asymptomatic ICH (median: 34.5 [IQR: 23.5–48.0] vs. 32.5 [26.0–56.2] min, respectively, p = 0.698).

### Diagnostic performance of the modified CLA test

ROC analysis indicated that the addition of cfDNA and histones to the assay mixture considerably improved the diagnostic performance of the CLA. Improvement of the diagnostic performance was most prominent for the 50%CLT parameter for the prediction of ICH (ROC AUC in the absence of DNA and histones: 0.56; 95%CI: 0.43–0.69; p = 0.371; ROC AUC in the presence of DNA and histones: 0.66; 95%CI: 0.54–0.78; p = 0.024). Of all test parameters, the CLA AUC parameter of the modified assay showed the best diagnostic performance measures to predict ICH (ROC AUC in the presence of cfDNA and histones: 0.69; 95%CI: 0.59–0.80; p = 0.006). Based on the optimal threshold value as defined by ROC analysis ( 29.9 OD * min), the CLA AUC parameter of the modified assay provided a remarkably high negative predictive value for the occurrence of ICH (97.9%; 95%CI: 92.7–99.8%) (Table 5). Regarding the prediction of short-term outcomes, the addition of cfDNA and histones improved the diagnostic parameters significantly, but the performance of the assay remained modest (ROC AUC for 50%CLT: 0.57; 95%CI: 0.49–0.65; p = 0.082; ROC AUC for 50%CLT in the presence of DNA and histones: 0.61; 95%CI: 0.53–0.69; p = 0.008).

**Table 5:** Diagnostic performance of most relevant clot lysis assay (CLA) parameters in the absence/presence of cell-free DNA and histones, according to short-term outcome and safety of thrombolysis.

Outcome	Parameter, unit	Threshold value	Specificity % (95%CI)	Sensitivity, % (95%CI)	NPV, % (95%CI)	PPV, % (95%CI)	LR (95%CI)	p
**CLA**								
Unfavorable short-term outcome/ no change	50%CLT, min	45.0	66.7 (56.3–76.0)	49.5 (39.1–59.9)	57.1 (47.5–66.5)	59.5 (47.9–70.4)	1.96 (1.09–3.51)	0.024
	CLA AUC, OD * min	27.7	62.5 (52.0–72.2)	52.6 (42.1–63.0)	57.1 (47.1–66.7)	58.1 (47.0–68.7)	1.85 (1.04–3.30)	0.036
ICH	50%CLT, min	39.0	56.8 (49.9–63.6)	61.1 (35.8–82.7)	94.5 (89.1–97.8)	10.7 (5.5–18.3)	2.07 (0.77–5.54)	0.142
	CLA AUC, OD * min	28.3	41.8 (35.1–48.7)	88.9 (65.3–98.6)	97.8 (92.3–99.7)	11.4 (6.7–17.9)	5.74 (1.29–25.6)	0.011
**CLA in the presence of cfDNA and histones (modified CLA)**								
Unfavorable short-term outcome/ no change	50%CLT, min	44.0	55.2 (44.7–65.4)	64.2 (53.7–73.8)	60.9 (49.9–71.2)	58.7 (48.6–68.2)	2.21 (1.34–3.95)	0.007
	CLA AUC, OD * min	25.4	44.8 (34.6–55.3)	73.7 (63.7–82.2)	63.2 (50.7–74.6)	56.9 (47.7–65.8)	2.27 (1.24–4.18)	0.007
ICH	50%CLT, min	39.0	62.0 (55.1–68.5)	66.7 (41.0–86.7)	95.7 (90.8–98.4)	12.9 (6.8–21.5)	3.26 (1.18–9.02)	0.017
	CLA AUC, OD * min	29.9	44.1 (37.4–51.1)	88.9 (65.3–98.6)	97.9 (92.7–99.8)	11.9 (7.0–18.5)	6.32 (1.42–28.17)	0.006

Diagnostic performance is provided for the optimal threshold value, as determined according to the Youden-index.

95%CI: 95% confidence interval; cfDNA, cell-free DNA; CLA, clot lysis assay; CLA AUC, clot lysis assay area under the curve parameter; 50%CLT, 50% clot lysis time; ICH, intracerebral hemorrhage; LR, likelihood ratio; NPV, negative predictive value; PPV, positive predictive value. (ROC analysis, χ^2^ test or Fisher’s exact test where appropriate).

A binary backward logistic regression model (including age, sex, increased NIHSS on admission, specific hemostasis/fibrinolysis proteins: fibrinogen, D-dimer, plasminogen activity, α2PI activity level and 50%CLT and CLA AUC parameters of the CLA in the absence or the presence of DNA and histones, based on Supplementary Table S1) revealed that a prolonged 50%CLT of the modified CLA (> 44 min) is a modest, independent predictor of recanalization failure as determined by the change in NIHSS by day 7 post-lysis (Table 6). On the other hand, in another regression model, a low CLA AUC parameter (< 29.9 OD * min) of the modified CLA proved to be a significant, independent predictor of post-lysis ICH (OR: 5.85; 95%CI: 1.24–27.7; p = 0.026). Besides this parameter, only NIHSS > 15 on admission remained in the stepwise backward regression analysis model as a significant, independent predictor of ICH (OR: 5.32; 95%CI: 1.69–16.75; p = 0.004).

**Table 6:** Independent predictors of poor short-term outcome and post-lysis intracranial hemorrhage in the studied cohort.

	OR	95%CI	p
**Poor short-term outcome**^**a**^			
50%CLT in the presence of cfDNA and histones, (> 44.0 min)	2.19	1.17–4.11	0.015
**Presence of post-lysis ICH**^**b**^			
NIHSS > 15	5.32	1.69–16.75	0.004
fibrinogen	1.52	1.00–2.284	0.050
CLA AUC in the presence of cfDNA and histones, (< 29.9 OD * min)	5.85	1.24–27.70	0.026

Last step of backward multiple regression analysis is provided.

cfDNA, cell-free DNA; CLA AUC, clot lysis assay area under the curve parameter; 95%CI, 95% confidence interval; 50%CLT, 50% clot lysis time; ICH, intracranial hemorrhage; NIHSS, National Institutes of Health Stroke Scale; OR, odds ratio.

^a^Poor short-term outcome is defined as a less than 4 points decrease or any increase of NIHSS by day 7 post-event, excluding patients with intracranial hemorrhage. Backward multiple regression model included age, sex, NIHSS on admission, BMI, fibrinogen, D-dimer, plasminogen activity, α2-plasmin inhibitor activity, 50%CLT (threshold: > 45 min) 50%CLT in the presence of cfDNA and histones (threshold: > 44 min), CLA AUC (threshold: > 27.7 OD * min), CLA AUC in the presence of DNA and histones (threshold > 25.4 OD * min).

^b^Backward multiple regression model included age, sex, NIHSS on admission, BMI, history of arterial hypertension, history of hyperlipidemia, fibrinogen, D-dimer, plasminogen activity, α2-plasmin inhibitor activity, 50%CLT (threshold: < 39 min), 50%CLT in the presence of cfDNA and histones (threshold: < 39 min), CLA AUC (threshold: < 28.3 OD * min), CLA AUC in the presence of DNA and histones (threshold: < 29.9 OD * min).

Another important utilisation of the modified CLA is that by choosing another threshold, it might be useful to predict which patients are not at risk of bleeding complications. This could be particularly useful in clinical scenarios when intravenous thrombolysis is administered before mechanical thrombectomy. In this cohort, when choosing the cut-off of 67 min of the 50%CLT of the mCLA, 46 patients (19.5% of the total cohort) could be distinguished as potential non-bleeders. When using the cut-off of 31.7 OD * min of the AUC parameter of the mCLA, 74 patients (32% of the total cohort) could be identified as potential non-bleeders, with 100% specificity.

## Discussion

Recent studies indicated a new model of stroke thrombus evolution, where, as the last step in the process of thrombi ageing, neutrophils infiltrate the thrombus by forming NETs and stabilize the thrombus with much smaller pores^23^. In fact, clot dissolution by rt-PA is the easiest in the early stages of thrombus formation, when the cross-linking of fibrin and fibrinolysis inhibitors to fibrin by activated factor XIII has not yet taken place, and the clot is less compact with larger pores. Although these events are likely to be crucial in the response to rt-PA, no hemostasis test exists that takes the effect of NET components into consideration. In this study, we demonstrate that a modified CLA supplemented with cfDNA and histones might be a promising tool to predict short-term outcomes and post-lysis intracerebral hemorrhagic complications in AIS patients undergoing i.v. thrombolysis. Moreover, when choosing a different threshold, the test might be useful to identify a considerable fraction of patients as potential non-bleeders. This could be an important aspect in clinical scenarios when thrombolysis is applied before mechanical thrombectomy. Recent guidelines propose that intravenous rt-PA should be considered for eligible patients even if mechanical thrombectomy is used^23–28^. To improve the safety of this approach, novel tests, such as the modified CLA might prove to be useful in the future.

Despite the clear benefit of diagnostic tests with acceptable predictive value regarding thrombolysis outcomes in AIS patients, surprisingly few studies are available on this topic. In a recent meta-analysis, where over 6400 records were screened, only four papers were found where hemostasis biomarkers were tested from a relatively large (> 100 patients) cohort of AIS patients before the start of reperfusion therapy^29^. Most studies collected blood samples within 24 h after stroke onset, which is a fairly wide interval. Ideally, a hemostasis biomarker of AIS thrombolysis outcome should be assessed before the initiation of treatment. Given the short time-window of i.v. thrombolysis, sample collection of relatively large cohorts could be a challenging task. In this study, we were able to enroll 231 AIS patients, all tested before thrombolysis and followed for specific outcomes and safety at days 1, 7 and 90 post-event.

Studies on predictive biomarkers of thrombolysis outcomes in AIS are often limited to investigating one or few hemostasis or fibrinolysis factors^9,29^. As the end result of thrombolysis is thought to depend on a sensitive balance and interaction between a series of factors and their inhibitors, the benefit of using a global assay for predicting therapy outcomes instead of measuring individual factors is biologically plausible. In particular, CLA is a theoretically optimal test for this purpose. As rt-PA concentrations used in this assay are much higher than endogenous t-PA concentrations, the CLA can be considered as a measure of fibrin resistance to therapeutical doses of exogenous rt-PA, rather than a marker of endogenous fibrinolytic capacity^30^. On the other hand, the CLA is a laborious test which suffers from several weaknesses. Firstly, it is poorly standardized, despite efforts to generate a standardized assay^31^. In our study, assay conditions were chosen based on available literature and a series of preliminary experimental conditions performed on healthy individuals. We optimized the assay conditions for a semi-automated testing of a relatively large set of patient samples, with an acceptable assay precision. Secondly, the assay is performed using plasma and therefore potential cellular contributors of thrombolysis resistance are not incorporated in the test. As an effort to improve the diagnostic performance of the assay, we supplemented the test with cfDNA and histones, mimicking the effect of NETs. It has been shown in several elegant studies that fibrin and NETs form a composite network within cerebral thrombi, and the effect of NETs is surmised to contribute to the overall lysability of clots in vivo^10^. Here we found that the addition of cfDNA and histones to the in vitro CLA mixture, as expected based on the literature, resulted in significantly prolonged clot formation and lysis^17,20,21^. Interestingly, the prolongation of clot lysis by cfDNA and histones showed inter-individual differences.

Significant increase in circulating cfDNA levels have been previously reported as a result of stroke-induced damage to the neurovascular unit in animal models and in few clinical studies as well^22,32–34^. In line with our report, cfDNA levels were found to be associated with stroke severity and post-stroke mortality in a handful of papers^22^. On the other hand, circulating cfDNA might not only be a stroke biomarker, but, in theory, potentially influence clot lysis. Our results showed that this is not the case as the cfDNA levels detected in this cohort did not show any association with conventional CLA parameters.

The presence of cfDNA within the thrombus, as a result of NETosis, however, could potentially influence lysis susceptibility and a test that imitates this effect might better predict treatment outcomes. Here we show that the diagnostic performance of the conventional CLA was considerably improved by the presence of cfDNA and histones in the assay mixture. It must be emphasized that the incorporation of cfDNA and histones to the test is an oversimplification of the effect of NETs within thrombi. The release of NETs is a finely tuned process that constitutes not only of the release of DNA and histones, but of other proteins, including neutrophil granule proteins (human neutrophil elastase, myeloperoxidase, etc.), leading to a variety of complex interactions within the thrombus^35^. Although the effect of cfDNA and histones are far from identical from the effect of intact NETs, DNA and histones have been shown to have important clot stabilizing and antifibrinolytic effects^17,36^. Among other mechanisms, cfDNA has been shown to accelerate tPA-PAI-1 complex formation, slow down t-PA mediated plasmin generation, modulate clot structure and delay plasmin-mediated lysis by intercalating into fibrin fibers^17,20,21,37,38^. Histones bind fibrinogen and fibrin and as a result of histone incorporation into polymerized fibrin, more stable clots are formed^21^. Conditions in these models and in our assay reflect pathologically high DNA concentrations that occur within thrombi. It is difficult to estimate the amounts of DNA that might be found in blood clots, but very high concentrations are likely as observed in previous studies^18,21^. In healthy individuals, cfDNA circulates at low levels (0.02–1.7 μg/ml) but elevated levels (5 μg/ml or above) have been detected in a variety of disease states, including sepsis^38^. In the modified CLA used in this study, optimal concentrations of DNA and histones were adapted from previous in vitro studies using purified fibrinogen and various concentrations of cfDNA and histones, testing their combined effect on fibrinolysis^21^. Although our primary goal was to find assay conditions where fibrinolysis kinetics are optimally influenced by the addition of cfDNA and histones, assay conditions of the modified CLA are likely to represent the increased pool of DNA and histones within the arterial thrombus, as published previously^18,21^.

Here we showed that results of the modified CLA was associated with short-term thrombolysis outcomes related to unsuccessful reperfusion. A prolonged (> 44 min) 50%CLT parameter of the modified assay was found to be a significant, independent predictor of therapy failure at 7 days. Significant association with short-term outcome was found only in case of the modified CLA, suggesting that the addition of cfDNA and histones to the assay mixture is crucial to obtain an improved diagnostic performance.

Furthermore, the modified CLA showed a high negative predictive value (97.9%) for the occurrence of ICH. Logistic regression analysis showed that the CLA AUC parameter of < 29.9 OD * min of the modified CLA is a significant, independent predictor of post-lysis ICH, similarly to increased NIHSS on admission. Intracerebral hemorrhagic complication is the most feared side-effect of rt-PA therapy, limiting its widespread use in less-experienced centers^39^. Nevertheless, only a handful of studies are available on hemostasis or fibrinolysis biomarkers predicting post-lysis ICH, as the number of patients with ICH in the investigated cohorts is often too low to draw any conclusions^29^. Risk models based on baseline characteristics have been shown to have limited clinical utility for improving thrombolysis safety^39^. Ideally, given the high negative predictive value of the modified CLA to predict ICH, it could be used to select those patients who are unlikely to have bleeding complications. Currently, the risk/benefit profile of thrombolysis prior to endovascular thrombectomy cannot be accurately predicted in individual cases, thus, incorporation of the CLA results into clinical predictive models could improve patient selection. Moreover, even in a case when the modified CLA yields increased ICH risk and the patient have already received thrombolysis within the shortest timeframe, the information could be relevant in the clinical practice, as the patient could be strongly monitored to reduce potential damage (e.g. longer ICU stay, aggressive control of hypertension, extensive neurologic follow-up, personalized post-lysis therapeutic approach, etc.). It must be noted, however, that our results did not show a difference between aSICH and SICH patients, which is most likely due to the fact that symptoms related to intracerebral bleeding are strongly influenced by the localization of the hemorrhage.

Results of the CLA on admission did not show an association with long-term functional outcomes (mRS at 3 months), which might be explained by the fact that the level of disability at 90 days post-lysis is driven by series of factors, often independent of the hemostasis/fibrinolysis system at the occurrence of the event (co-morbidities, socio-economic status, post-event infections, stc.). Notably, the 50%CLT parameter of both assay conditions showed a significant, step-wise negative association with stroke severity (a major determinant of long-term functional outcomes). The negative association between stroke severity and 50%CLT is a puzzling result that was not associated with significant consumption of the few individual hemostasis and fibrinolysis proteins measured to test this possibility. A non-significant trend for lower α2-PI activity was found in more severe strokes, which could be in line with the observed data, but other fibrinolytic proteins of relevance might have a yet uncovered effect in this respect. Interestingly, plasminogen activity also showed a significant, step-wise negative association with stroke severity in this cohort- the biological relevance of which needs to be investigated in further studies.

In conclusion, our data implicate that the modified CLA using pre-thrombolysis plasma of AIS patients might be a useful tool to predict short-term outcomes and post-lysis intracerebral hemorrhagic complications after i.v. rt-PA therapy. It must be emphasized, that our findings require external validation. The modified test supplemented with cfDNA and histones as described here could represent a starting point to further improve the CLA to reach optimal diagnostic performance, assay precision and shorter, potentially automated execution.

## Materials and methods

### Patients

In this observational study, AIS patients were enrolled in a single stroke center (Department of Neurology, University of Debrecen, Hungary). Patient enrollment started in September 2016 and finished in April 2019. Inclusion and exclusion criteria of patients were identical to the standard criteria of rt-PA administration according to 2008 ESO guideline^40^. All patients underwent thrombolysis within the 4.5 h therapeutic time window using rt-PA (Boehringer Ingelheim, Germany) according to standard protocols^40^. Patients receiving mechanical thrombectomy in addition to thrombolysis were not included in the study. The presence of AIS was diagnosed based on clinical symptoms, brain imaging using non-contrast computerized tomography (CT) scan, and CT angiography (CTA). A control CT was performed for every patient 24 h after the event. CT images taken on admission and 24 h post-lysis were analyzed simultaneously by 3 independent investigators and the Alberta Stroke Program Early CT Scores (ASPECTS) were calculated^41^. For each patient, the time of symptom onset, demographic and clinical characteristics (age, sex, BMI, previous medications, history of cerebrovascular and cardiovascular diseases, cerebrovascular risk factors including smoking) were registered on admission. Stroke severity was determined by the National Institutes of Health Stroke Scale (NIHSS)^42^ on admission and day 7 after therapy. Trial of ORG 10172 in Acute Stroke Treatment (TOAST) criteria was used to identify the etiology of stroke^43^. Patients were followed and long-term functional outcomes were determined at 3 months after the stroke event using the modified Rankin Scale (mRS)^44–47^.

The following outcomes and safety endpoint were registered: (1) Short-term outcome at 7 days post-event: a decrease in NIHSS score by at least 4 points or to 0 was defined as favorable outcome, while an increase in NIHSS score by at least 4 points was defined as unfavorable outcome^5,48^. (2) Long-term outcome at 90 days post-event: mRS 0–1 was defined as favorable long-term outcome^47^. (3) Hemorrhagic transformation: symptomatic (SICH) or asymptomatic intracranial hemorrhage (aSICH) using the European Cooperative Acute Stroke Study (ECASS) II criteria^49^.

### Informed consent

The study design was in accordance with the guiding principles of the Declaration of Helsinki and was approved by the Institutional Ethics Committee of the University of Debrecen and the Ethics Committee of the National Medical Research Council. All patients or their relatives provided written informed consent.

### Blood sampling and laboratory measurements

Peripheral blood samples were taken from all patients on admission, before the initiation of rt-PA infusion. Laboratory examinations were carried out based on standard procedures in our laboratory, according to manufacturers’ instructions, as reported previously^50,51^. Briefly, routine laboratory tests (ions, glucose level, renal and liver function tests, high-sensitivity C-reactive protein measurement, complete blood count) were carried out immediately by standard laboratory methods (Roche Diagnostics, Mannheim, Germany and Sysmex Europe GmbH, Hamburg, Germany). For the examination of hemostasis tests and cfDNA, blood samples were collected to vacutainer tubes containing 0.109 M sodium citrate (Becton Dickinson, Franklin Lane, NJ) and were processed immediately to gain platelet free plasma (centrifugation twice at 1500*g*, room temperature for 15 min). Screening tests of coagulation (prothrombin time, activated partial thromboplastin time, and thrombin time) were performed immediately on a BCS coagulometer using routine methods (Siemens Healthcare Diagnostic Products, Marburg, Germany). For the execution of in vitro CLA, other specific hemostasis tests, and cfDNA, aliquots of citrated plasma were labeled with a unique code and stored at − 80 °C until analysis. In vitro CLA, specific hemostasis assays and determination of cfDNA were performed by investigators blinded to patient identification and clinical data.

### Measurement of cell-free DNA (cfDNA)

The method of cfDNA measurement was carried out according to previous reports^22,52^. Briefly, plasma cfDNA levels were quantified using fluorescent nucleic acid stain, Quant-iT PicoGreen dsDNA reagent and kit assay (Thermo Fisher Scientific, Waltham, Massachusetts, USA) according to the manufacturer’s instructions. Briefly, a 5-point standard from 1 ng/mL to 1 μg/ml was prepared by serial dilutions. Standards and samples (100 μl) were loaded into black 96-well plates (Greiner Bio-One, Kremsmünster, Austria) followed by the addition of 100 μl working solution of the Quant-iT PicoGreen reagent to each sample, incubation for 5 min at room temperature, protected from light. The fluorescence intensity was quantified at 480 nm using a TECAN Infinite p200 PRO microplate reader (TECAN Trading AG, Männedorf, Switzerland).

### In vitro clot lysis assays

Recombinant t-PA-driven lysis of tissue factor-induced plasma clots was studied in 96-well microtiter plates by monitoring changes in turbidity. Final assay conditions were set based on previous studies, with some modifications, optimized for reliable high-throughput analysis of patient samples^30,31,53–55^. Two assay conditions were used, and plasma samples were run in quadruplicates in both assay conditions. All concentrations provided refer to final concentrations in the 100 μL final well volume. Plasma samples were thawed in a water bath at 37 °C. In the first assay condition, a clot induction and lysis mix was prepared, where citrated plasma was mixed with 1000-fold diluted human tissue factor (Innovin, Siemens, Marburg, Germany) and 100 ng/ml rt-PA (Alteplase, Boehringer Ingelheim, Ingelheim, Germany) in HEPES buffer (10 mM HEPES, 150 mM NaCl, 0,05% Tween20, pH:7.4). In another set of experiments, in order to imitate the effect of NETs, 150 μg/ml pure and cell-free DNA (cfDNA; calf thymus DNA, Sigma-Aldrich, Darmstadt, Germany) and 50 μg/ml calf thymus histone (TIII S, Calbiochem, La Jolla, CA, USA) were added to the clot induction and lysis mixtures. Optimal concentrations of cfDNA and histones were adopted based on previous literature where the combined effect of histones (50 μg/ml) and various concentrations of cfDNA (50–250 μg/ml) were studied on fibrinolysis kinetics in purified experimental conditions^21^. Dilution of plasma samples with buffer was 1.2-fold. Clotting and subsequent lysis were induced with automated sample pipetting of HEPES buffer, containing 21 mM CaCl_2_, to each sample well. Optical density was measured at 340 nm, 37 °C every minute for 300 min in a TECAN Infinite m200 microplate reader (TECAN Trading AG, Männedorf, Switzerland). Curves were analyzed using the Shiny app software tool^56^. Clot formation and lysis were defined using the following variables calculated from the turbidimetric curves: maximum absorbance, time to maximum absorbance, various points of clot lysis time (CLT): 10% clot lysis time (10%CLT), 50%CLT, 90%CLT and area under the curve (CLA AUC). Clot lysis times were defined as the time from the 10%, 50% or 90% point, from clear to maximum turbidity, to the 10%, 50% or 90% point in the transition from maximum turbidity to the final baseline turbidity, respectively (resulting in 10%CLT, 50%CLT and 90%CLT parameters, respectively). Analytical precision of both assay conditions was evaluated according to the guidelines of Clinical and Laboratory Standards Institutes (CLSI document EP05-A3)^57,58^. Precision was tested using healthy control plasmas, each run in quadruplicate, for 20 days. Coefficients of variation (CVs) of the within-run and total (within-laboratory) precision assessments were 8.6% and 8.9%, respectively. Precision results were essentially similar in both assay conditions.

### Specific hemostasis assays

Specific hemostasis assays were measured according to standard procedures in our laboratory, as described previously, based on the manufacturers’ recommendations^51,59,60^. Quantitative D-dimer levels were measured using a particle-enhanced, immuno-turbidimetric assay (Innovance D-dimer) on a BCS coagulometer according to the manufacturer’s instructions (Siemens Healthcare Diagnostic Products, Marburg, Germany). α2-plasmin inhibitor (α2-PI) activity and plasminogen activity were determined by commercially available methods (Siemens Healthcare Diagnostic Products, Marburg, Germany). Fibrinogen levels were analyzed according to the method of Clauss using standard methods.

### Statistical analysis

Statistical analysis was performed using the Statistical Package for Social Sciences (SPSS, Version 26.0, Chicago, IL), and GraphPad Prism 8.0 (GraphPad Prism Inc., La Jolla, CA). The study was powered to have a 90% chance of detecting 10% true difference between two subgroups, setting the value of α (type I error rate) to 0.05, based on previous CLA assay results^61^. To demonstrate such or greater difference, a minimum of 18 patients were required per group. Shapiro–Wilk test was used to assess the normality of the data. Student’s t test or Mann–Whitney U test was performed for two-group analyses. In case of paired data, paired t-test or Wilcoxon signed-rank test was applied. ANOVA with Bonferroni post hoc test or Kruskal–Wallis analysis with Dunn–Bonferroni post hoc test was applied for multiple comparisons. Pearson’s or Spearman’s correlation coefficient was used to determine the strength of correlation between continuous variables. Differences between categorical variables were assessed by χ^2^ test or by Fisher’s exact where appropriate. Receiver operating characteristic (ROC) curves were built by plotting sensitivity vs. 1-specificity and calculating the area under the curve (AUC). Optimal threshold values of the CLA parameters were calculated based on Youden’s J statistics. Test characteristics of sensitivity, specificity, positive predictive value (PPV), and negative predictive value (NPV) were calculated using contingency tables and χ^2^ test or Fisher’s exact at statistically optimal threshold values. Binary backward logistic regression models were used to determine independent predictors of short-term functional outcome and therapy-associated intracerebral bleeding. Adjustments of the models were based on the results of preliminary statistical analyses of baseline characteristics between groups (Student’s t test or Mann–Whitney U test, χ^2^ test or Fisher’s exact), previous literature, and methodological principles (dichotomized variables when possible). Results of the logistic regression analysis were expressed as odds ratio (OR) and 95% confidence interval (CI). A p-value of < 0.05 was considered statistically significant.

## Limitations

Results of the present study should be interpreted in the context of its limitations and strengths. The sample size is limited, however, as compared to other published studies measuring hemostasis or fibrinolysis biomarkers in stroke patients from pre-thrombolysis samples, it is the largest study as yet^29^. Due to the limited number of patients with post-lysis ICH, despite the significant associations found, results presented here must be confirmed and validated by larger studies. Being single-centered, our study had the advantages of uniform sample handling and uniform patient care, but, as the center recruits patients from a relatively large geographic area, unfortunately, a proportion of patients were lost to follow-up due to the transfer of patients (9.1% and 8.7% for short-term and long-term follow up, respectively). This percentage of follow-up drop-out is comparable or even lower to that observed in other studies involving post-stroke patients^29^, however, it might have influenced the results to a certain extent and thus larger clinical studies are needed to confirm and to validate our data.

## Supplementary Information

Supplementary Information.
