# Supplementary material for: A modified in vitro clot lysis assay predicts outcomes and safety in acute ischemic stroke patients undergoing intravenous thrombolysis
Source: Sci Rep. 2021 Jun 16;11:12713. doi: 10.1038/s41598-021-92041-1 (PMC8208992; doi:10.1038/s41598-021-92041-1)
Supplement: Supplementary file 1 — Supplementary Information. [file 41598_2021_92041_MOESM1_ESM.docx]

**Supplementary Table S1. Admission clot lysis assay (CLA) parameters and specific hemostasis/fibrinolysis protein levels according to short-term functional outcomes at 7 days post-event**

|  | **Favorable outcome**  **(n=97)*** | **No change/**  **unfavorable outcome**  **(n=95)*** | **p**  **p** |
| --- | --- | --- | --- |
| CLA | | | |
| maximal absorbance | 1.4±0.2 | 1.4±0.2 | 0.355 |
| time to maximal absorbance (min) | 11.1  (9.0-14.3) | 10.5  (8.0-18.0) | 0.899 |
| 10%CLT (min) | 31.0 (19.0-39.8) | 31.0 (22.0-42.4) | 0.285 |
| 50%CLT (min) | 40.5 (27.0-53.8) | 45.3 (31.3-60.5) | 0.062 |
| 90%CLT (min) | 69.0 (54.4-100.8) | 82.5 (60.0-111.0) | 0.090 |
| CLA AUC (OD*min) | 26.0 (21.2-30.9) | 28.4 (23.4-33.7) | 0.082 |
| CLA in the presence of cfDNA and histones (modified CLA) | | | |
| maximal absorbance | 1.4±0.2 | 1.4 ±0.2 | 0.579 |
| time to maximal absorbance, (min) | 11.3  (9.0-17.0) | 11.3  (9.0-17.3) | 0.604 |
| 10%CLT, (min) | 32.3 (21.0-48.5) | 37.0 (24.8-52.6) | 0.107 |
| 50%CLT, (min) | 40.5 (29.0-58.8) | 50.3 (36.0-69.0) | 0.007 |
| 90%CLT, (min) | 81.0 (57.0-102.0) | 91.1(66.0-112.0) | 0.065 |
| CLA AUC, (OD*min) | 27.0 (21.7-33.6) | 30.3 (24.4-35.5) | 0.036 |
| Specific hemostasis/fibrinolysis proteins | | | |
| fibrinogen (g/L) | 4.0 (3.0-4.7) | 4.1 (3.5-4.7) | 0.858 |
| D-dimer (mg/L) | 0.8 (0.5-1.5) | 0.7 (0.5-1.1) | 0.134 |
| plasminogen activity (%) | 95.0 (84.0-108.0) | 100.0 (91.0-111.0) | 0.053 |
| α2-PI activity (%) | 102.0 (90.0-110.0) | 103.0 (95.0-112.0) | 0.426 |

Data are means±SD or medians (interquartile ranges). α2-PI, α2-plasmin inhibitor; cfDNA, cell-free DNA; CLA, clot lysis assay; 10%CLT, 10% clot lysis time; 50%CLT, 50% clot lysis time; 90%CLT, 90% clot lysis time; CLA AUC, clot lysis assay area under the curve. * excluding patients with ICH.

**Supplementary Table S2. Admission clot lysis assay (CLA) parameters and specific hemostasis/fibrinolysis protein levels according to long-term functional outcomes at 90 days post-event**

|  | **mRS 0-1**  **(n=104)*** | **mRS 2-6**  **(n=89)*** | **p** |
| --- | --- | --- | --- |
| Clot lysis assay (CLA) | | | |
| maximal absorbance | 1.4±0.2 | 1.4±0.2 | 0.207 |
| time to maximal absorbance (min) | 11.6 (9.0-15.0) | 10.0 (8.0-16.0) | 0.476 |
| 10%CLT (min) | 31.0 (22.0-40.0) | 27.0 (19.0-42.0) | 0.439 |
| 50%CLT (min) | 42.0 (32.0-59.0) | 37.1 (27.3-58.8) | 0.288 |
| 90%CLT (min) | 72.0 (58.0-110.3) | 77.0 (54.4-107.0) | 0.904 |
| CLA AUC (OD*min) | 26.9 (22.4-32.0) | 25.5 (21.2-31.8) | 0.331 |
| Clot lysis assay in the presence of cell-free DNA and histones (modified CLA) | | | |
| maximal absorbance | 1.4±0.2 | 1.4±0.2 | 0.095 |
| time to maximal absorbance, (min) | 11.3(9.0-19.0) | 12.0 (8.0-18.0) | 0.549 |
| 10%CLT, (min) | 40.7 (24.3-50.8) | 30.0 (21.4-42.0) | 0.138 |
| 50%CLT, (min) | 48.5 (34.3-64.0) | 43.8 (30.2-66.9) | 0.416 |
| 90%CLT, (min) | 78.0 (57.9-111.0) | 87.0 (65.0-108.0) | 0.724 |
| CLA AUC, (OD*min) | 28.8 (23.9-33.9) | 27.8 (22.5-33.8) | 0.352 |
| Specific hemostasis/fibrinolysis proteins | | | |
| fibrinogen (g/L) | 4.1 (3.4-4.7) | 4.3 (3.7-5.0) | 0.024 |
| D-dimer (mg/L) | 0.7 (0.5-1.2) | 0.9 (0.5-2.0) | 0.010 |
| plasminogen activity (%) | 99.0 (88.0-111.0) | 95.0 (86.0-107.0) | 0.173 |
| α2-PI activity (%) | 105.0 (93.0-111.0) | 104.0 (94.0-109.0) | 0.456 |

Data are means±SD or medians (interquartile ranges); α2-PI, α2-plasmin inhibitor; 10%CLT, 10% clot-lysis time; 50%CLT, 50% clot-lysis time; 90%CLT, 90% clot-lysis time; CLA AUC, clot lysis assay area under the curve; mRS, modified Rankin Scale; * excluding patients with ICH. (unpaired Student’s t test, Mann-Whitney U test)
